# Report of the Eighth General Meeting of the Sydenham Society, Held at the Society’s Rooms, on Wednesday, May 1st, 1850

**Published:** 1851-02

**Authors:** 


### BIBLIOGRAPHICAL NOTICES.

*Report of the Eighth General Meeting of the Sydenham Society, held at the Society's rooms, on Wednesday, May 1st,* 1850. SIR JAMES CLARK, Bart. M. D., F. R. S., President of the Society, in the chair. London: C. & J. Adlard.

We have repeatedly urged upon the readers of this Journal the importance of the ancient literature of the medical profession, and we have on several occasions given illustrations of the value of that literature by extracts from it. Those who may plunge deeply into it, will find rich pearls in its quiet depths, and they will learn to place a proper estimate upon those lapidaries, who, under pretence of having found new gems, make no ordinary stir in the world, when, in truth, they have merely cut off a brilliant from its parent stock. There is a healthy, vigorous stream of common sense, a rich vein of fruitful experience, pervading the works of the old masters, that should not be slighted by any one who wishes to stand fair in what may be read in the medical profession. The hardy pearl-diver finds rich gems occasionally in the most frequented spots, and may grow rich by diligent search of neglected places. Even Cuvier, rich in the lore of the past, undertook certain anatomical investigations under the firm persuasion that he was the first in the path, until he looked into the works of Aristotle, and found that he had been completely anticipated by the acute, and universally learned Stagirite, and if Cuvier could receive instruction from Aristotle, it is not impossible but that some of us might stumble on something useful in the works of Hippocrates and Paulus Ægineta. It is improving to look into their works, at least to find out what they knew; to see how far they advanced the healing art with their imperfect means, and how far their methods of observation and of accumulating experience may be trusted now. There is no luxuriance of merit in a flippant ignorance of their teachings.

About eight years since, a number of Medical gentlemen in England, who were imbued with a just appreciation of the value of the labors of some of the ancient cultivators of medical science, united themselves as a Society, which they properly called the Sydenham Society. Their object was not only to rescue the venerable oracles of ancient times from oblivion, but to do it in such a way as to make them accessible to the medical public. Thus far the labors of this Society have been eminently serviceable, and deserve encouragement. The object of the Sydenham Society is to "meet certain acknowledged deficiencies in existing means for diffusing medical literature, which are not likely to be supplied by the efforts of individuals." The Society's publications have been distributed among 2,543 members of the profession. Those who have been members from the beginning of the Society have now received for their seven annual subscriptions of £1 1s, twenty-three 8 vo. volumes, published in excellent style, comprising 11,000 very full pages of letter-press, and twenty-six illustrative plates. "The ordinary publishing price of the Sydenham Society's works would probably be about £16 or £17."

The literary objects of the Sydenham Society are comprised under the following heads:—I. Reprints of standard English works—such as those of Sydenham, Harvey, and Hewson. II. Miscellaneous Selections from the ancient and from the earlier modern Authors, such as "Observations on Aneurism," "Memoirs of the French

Academy of Surgery," and "Essays on Diseases peculiar to women," works which were inaccessible to the body of the profession. III. Translations of the Greek and Latin medical authors, and of works in the Arabic and other eastern tongues"—such as "seven books of Paulus Ægineta," "The genuine works of Hippocrates," and the "Treatise of Rhazes on the small pox." IV. Translations of recent Foreign Works of merit." This department of publication, by the Sydenham Society, has been more voluminous than any other, and the works are very valuable.

Another important object of the Society is the publication of "original works of merit, which might prove valuable as books of reference, but which would not otherwise be published, from the slender chance of their meeting with a remunerative sale, such as Bibliographical, alphabetical and digested indices to voluminous periodical publications. Of the great value of works of this kind, there can be no kind of doubt, and we have recently understood that the Sydenham Society has secured several valuable manuscripts of the kind referred to. The intention of the Society is to make a Medical Bibliography, which "will not be a mere dry catalogue of books; but, whilst indicating every source whence information on any branch of medical knowledge may be obtained, it will give such historical and biographical notices, as will make the work of interest and value even to those who may not use it for the ordinary purposes of bibliography. The Council have already secured the means for supplying, in a complete form, the Medical Bibliography of all countries, from the earliest period down to the revival of letters in the sixteenth century, and the entire Medical Bibliography of this country, down to the close of the last century."

Among other works in preparation are "a volume on epidemic Catarrh," "the writings of Unzer and Prochaska on the Nervous system," "a further selection from the works of Dupuytren," "a volume to comprise the most important writing of the earlier British Physiologists," and "an edition of Hunter's Plates of the Gravid Uterus."

We earnestly wish that physicians throughout this country would encourage the enterprise of the Sydenham Society. There is not a village in the Union that does not contain physicians enough to secure the works of the Society by clubbing. They could easily enter into arrangements for the safe-keeping of the books, and for the proper use of them. And while encouraging the labors of the Sydenham Society, these physicians, far removed from access to large medical libraries, would secure the means of improving themselves in their professional knowledge and skill, and greatly benefit their patients. The cost is too ridiculously small to make an impediment to the success of the scheme. Something should be done to rouse the profession to the importance of reading sound medical literature, for that is the only method we know of for erecting a bulwark against quackery, upon which charlatanism may send its surges in vain. Whenever the entire profession devotes itself assiduously to study; when the public feels confidence in the depth and breadth of professional knowledge, and feels secure that it is up to the mark in all the requirements of science, then, and not till then, can we expect an enlarged and an abiding confidence in professional abilities. It is almost incredible to think of the hours that should be devoted to communion with the excellent minds of the past and present, that are thrown away upon frivolity, or wasted in sleep. It is shocking to think of the perfection of the means for making sluggish brains dull, and of the little care that is manifested for those adornments of the mind that will survive the tomb, and flourish in eternal youth.

We cannot but believe that if physicians were more careful in the cultivation of social intercourse with one another, there would be a greater and more commanding harmony than is often found. There is nothing more destructive of petty jealousies, ill-founded prejudices, and snarling invectives than a free and cheerful interchange of the amenities and comities of life. And there is nothing better calculated to encourage the latter, than improvement at the same fountain-heads of knowledge. There are paltry things in the profession, who do what little flourishing they exhibit, through the general ignorance that prevails, but in the midst of well directed cultivation of the intellect, they would soon stumble upon their native nothingness, and die as the fool dieth. Isaachar is an ass crouching between two burdens,—the desire to be something, and the inability to be; he is indifferent to the crib, and is hopeful of provender.

We pray the attention of the readers of these remarks to the terms of the Sydenham Society. Three or four physicians may easily unite in procuring the works published by the Society. They can agree upon the terms for the common use of the books, which may be placed in the custody of one of the subscribing club as librarian. In this way those physicians may not only do themselves, but the community in which they practice, an incalculable service.

The following works have been already published:

FOR THE FIRST YEAR, 1843–'4.

Hecker's Epidemics of the Middle Ages, one Vol. 8vo, pp. xx, 380.
Louis on Phthisis, one Vol. 8vo, pp. xxxv, 571.
Th. Sydenham Opera Omnia, one Vol. 8vo, pp. xxx, 668.

FOR THE SECOND YEAR, 1844–'5.

The Seven Books of Paulus Ægineta, Vol. I, pp. xxviii, 683.

Observations on Aneurism, one Vol. 8vo, pp. xii, 524.
Simon's Animal Chemistry, Vol. I, 8vo, pp. xx, 360, plate.

FOR THE THIRD YEAR, 1845–'6.

Simon's Animal Chemistry, Vol. II, 8vo, pp. xii, 560, two plates.
Paulus Ægineta, Vol. II, 8vo, pp. xi, 511.
Hasse's Pathology, 8vo, pp. xvi, 400.

FOR THE FOURTH YEAR, 1846–'7.

The Works of W. Hewson, in one Vol. 8vo, pp. lvi, 360, portrait and eight plates.
Dupuytren's Lectures on Diseases and Injuries of Bones, one Vol. 8vo, pp. xvi, 459.
The Works of W. Harvey, M.D., complete in one Vol. 8vo, pp. xcvi, 624.

FOR THE FIFTH YEAR, 1847–'8.

Paulus Ægineta, Vol. III, pp. viii, 653.
Feuchtersleben's Medical Psychology, pp. xx, 362.
Microscopical Researches of Schwann and Schleiden, pp. xx, 268, six plates.
Memoirs of the French Academy of Surgery, pp. x, 293.

FOR THE SIXTH YEAR, 1848–'9.

Rhazes on the Small Pox and Measles, 8vo, pp. viii, 212.
The Genuine Works of Hippocrates, translated from the Greek, Vol. I, 8vo, pp. x, 466.
The Works of Sydenham, translated from the Latin, Vol. I, 8vo, pp. cvi, 276.
Rokitansky's Pathological Anatomy, Vol. II, (the first issued), 8vo, p. 375.

FOR THE SEVENTH YEAR, 1849–'50.

The Genuine Works of Hippocrates, Vol. II, pp. 406.
Essays on the Puerperal Fever, and other Diseases peculiar to Women, 8vo, pp. 552.
The Works of Sydenham, translated from the Latin, Vol. II, pp. 396.

*Notice.*—New Members may still obtain the 1st, 4th, 5th, and 6th year's works; but of the 2d and 3d year's books none remain. In lieu of the third volume of *Paulus Ægineta,* new members will receive a copy of some other work, so that on commencing with the fourth or either of the subsequent years, the series of books which they will obtain, will be complete in themselves.

The leading physicians of England are either officers or members of the Sydenham Society, and the publications are made under the advice and superintendance of the ablest minds in Great Britain. Among the officers of the society are local secretaries, through whom a large share of the business is done. The local secretary for the United States is Professor Dunglison, of Philadelphia. Through him, subscribers in this country may obtain their packages by paying him a due share of the carriage of the parcel in which the books are sent.

"Those members who wish their books to be included in any of the parcels to Local Secretaries, are requested to send in their names, with their Christian name in full, and particular address to the various Local Secretaries, who are respectfully requested to forward to the Secretary for London an accurate list of all such names with as little delay as possible."

Among the laws of the Sydenham Society are the following:

"The subscription constituting a member shall be *one guinea;* to be paid in advance on the 25th day of March, annually; for which he shall be entitled to a copy of every work published by the Society for the year for which he subscribes. Any subscriber who shall not have paid his subscription previous to the last of July of the current year, shall not be allowed the privileges of that year's subscription except on the payment of a fine of five shillings; nor shall any one be allowed to join the Society for the current year after that period except on payment of a similar fine.

"The Council shall lay before the members, at each anniversary meeting, a report of their proceedings during the past year, and also an account of the receipts and expenditures of the Society; and shall further cause to be printed and circulated among the members an abstract of such report and accounts immediately after each anniversary meeting.

"Members shall have the privilege of proposing works for publication, and shall address their propositions to the Council.

"The works of the Society shall be handsomely printed, on a uniform plan, for members only.

25th Law.—"It shall not be competent for book-clubs to subscribe to the Society as members; but it shall be permitted to societies and institutions having *permanent* libraries, to subscribe to the Society in the name of their President, Secretary, or Librarian, or other responsible officer."

In reference to the 25th law, it is obvious that any one of the members of the book club may be chosen as the one in whose name the subscription may be made. We indulge the hope that the laudable enterprize of the Sydenham Society will command the favor and encouragement of a large portion of the medical profession in this country. B.

